# Surgery vs. non-surgery for advanced cholangiocarcinoma post-conversion therapy with PD-1/PD-L1 inhibitors plus TKIs

**DOI:** 10.3389/fimmu.2026.1753437

**Published:** 2026-01-29

**Authors:** Zengpeng Sun, Yutao Wang, Xu Chen, Lishun Yang, Ou Li, Jia Zhou, Zhiguo Tan, Chuang Peng

**Affiliations:** Department of Hepatobiliary Surgery, Hunan Provincial People’s Hospital/The First Affiliated Hospital of Hunan Normal University, Changsha, China

**Keywords:** conversion therapy, PD-1/PD-L1 inhibitors, surgical treatment, TKIs, unresectable cholangiocarcinoma

## Abstract

**Objective:**

To compare overall survival (OS) and progression-free survival (PFS) between surgical resection and non-surgical therapy in initially unresectable Cholangiocarcinoma (CCA) patients who achieved radiologic/MDT-confirmed resectability after PD-1/PD-L1 inhibitors plus TKIs.

**Methods:**

We performed a retrospective analysis of 47 patients with initially unresectable CCA admitted between June 2020 and December 2024. Based on post-conversion treatment strategies, patients were divided into non-surgical resection (NR) and surgical resection groups (SR). We collected detailed baseline clinical data, treatment-related parameters, and long-term survival outcomes for all participants. Overall survival (OS) and progression-free survival (PFS) were compared between groups, with recurrence-free survival (RFS) analyzed in the SR. Cox regression was used to identify prognostic risk factors.

**Results:**

This study enrolled a total of 47 patients, including 23 in the SR and 24 in the NR. No significant differences were observed in baseline data between the two groups before conversion therapy. In the SR, the median overall survival (OS) was not reached, with 1-, 2-, and 3-year OS rates of 95.7%, 68.5%, and 68.5%, respectively. These survival outcomes were significantly superior to those observed in the NR, where the median OS was 28.5 months, and the 1-, 2-, and 3-year OS rates were 91.7%, 51.4%, and 17.6%, respectively (P = 0.026). Additionally, the SR exhibited a significantly longer median progression-free survival (PFS) of 19 months, with corresponding 1-, 2-, and 3-year PFS rates of 87.0%, 40.2%, and 25.2%. In contrast, the NR had a median PFS of 13.5 months and 1-, 2-, and 3-year PFS rates of 61.6%, 12.4%, and 12.4%, respectively (P = 0.025). Among patients in the SR, 21 cases (91.3%) achieved R0 resection, with no surgery-related mortality reported. The 1-, 2-, and 3-year recurrence-free survival (RFS) rates in this subgroup were 54.7%, 39.0%, and 29.3%, respectively.

**Conclusion:**

For patients with initially unresectable CCA, PD-1/PD-L1 inhibitors plus TKIs can successfully downstage the tumor. Conversion surgery is safe and feasible, and surgical treatment can improve patients’ OS and PFS.

## Introduction

Cholangiocarcinoma (CCA) is a malignant tumor with marked heterogeneity that derives from biliary epithelial cells. Anatomically, this cancer is categorized into three subtypes: intrahepatic cholangiocarcinoma (iCCA), perihilar cholangiocarcinoma (pCCA), and distal cholangiocarcinoma (dCCA). Globally, the incidence of CCA has exhibited a sustained upward trend (1). As one of the malignant tumors with the worst prognosis in the digestive system, CCA is characterized by strong occultation, high invasiveness, and difficulty in early diagnosis. A large proportion of patients are identified with advanced local disease or distant metastases upon diagnosis, thus losing the opportunity for curative surgical resection (2). For patients with initially unresectable CCA, the prognosis is extremely poor. In 2010, the ABC-02 study recommended the gemcitabine + cisplatin (GC) regimen as a first-line treatment option for advanced CCA and reported an ORR of 26.1% (3). However, conventional chemotherapy regimens such as GC yield a median overall survival (OS) of less than 12 months and a 5-year OS rate of below 10% (4, 5), which brings severe challenges to the clinic and urgently needs to explore a better treatment scheme.

In recent years, development of tumor molecular biology and immunotherapy technology, the therapeutic landscape of CCA has undergone a revolutionary changes. Precision interventions of molecular targeted therapy against tumor-specific driver genes, together with the use of immune checkpoint inhibitors (ICIs) as monotherapy or combination regimens, have significantly improved the objective response rate (ORR) and survival benefits of patients diagnosed with unresectable CCA (6–9). What’s more noteworthy is that targeted/immunotherapy-based regimens have enabled a subset of patients to achieve tumor downstaging or radiologically confirmed resectable conversion, thereby unlocking new possibilities for curative surgical intervention. This “conversion therapy” strategy has now become a core research focus in advanced CCA management (10, 11). The synergistic combination of PD-1/PD-L1 inhibitors and TKIs has emerged as a rational and effective conversion therapy strategy for CCA, primarily due to complementary anti-tumor mechanisms. Validated in real-world and clinical studies (12–14), this dual-action approach yields superior tumor downstaging rates and objective response rates (ORR) compared to chemotherapy alone or single-agent immunotherapy in advanced CCA, making it an optimal conversion platform. However, there is a key clinical conundrum that needs to be addressed: in initially unresectable CCA patients who attain successful conversion through targeted or immunotherapy, is curative surgical resection or continued targeted/immunotherapy more beneficial for survival? This question remains unresolved to date. On one side, as the only potentially curative modality, surgery can theoretically completely eradicate residual tumor tissue and reduce the risk of recurrence. However, surgery following conversion therapy is confronted with multiple challenges, such as adhesion between tumor tissue and surrounding normal organs, increased degree of vascular invasion, and elevated incidence of postoperative complications. For some patients, surgical trauma may lead to diminished quality of life or even perioperative mortality. On the other hand, continuous non-surgical treatment can avoid surgery-related risks and control residual tumors through long-term pharmacological intervention. However, existing studies have shown that even in patients responding to chemotherapy or targeted/immunotherapy, long-term maintenance therapy can hardly avoid the development of drug resistance, which may ultimately lead to disease progression. Additionally, there is a lack of survival breakthroughs brought by curative modalities (15–17).

A recent study (18) demonstrated that for initially unresectable biliary tract cancer (BTC), those who underwent surgical treatment after achieving successful conversion with PD-1/PD-L1 inhibitor-based therapy achieved superior OS and PFS. However, this study has limitations. Owing to its retrospective nature and limited sample size, the external validity of its results is constrained. Nevertheless, considering the rapid advancement in immunotherapy for advanced BTC, a preliminary investigation of the feasibility, curative effect, and safety profile of conversion surgery is highly meaningful. Therefore, we aimed to address whether surgical resection improves survival versus continued non-surgical therapy in initially unresectable CCA patients achieving radiologic/MDT resectability post-PD-1/PD-L1 + TKIs. We hypothesized that, among such patients, those undergoing surgical resection would have better OS and FPS than those continuing non-surgical systemic therapy.

## Materials and methods

### Study design and patients

Patients with histologically confirmed initially unresectable CCA admitted to Hunan Provincial People’s Hospital between January 2020 and December 2024 were retrospectively screened. The unresectability was comprehensively determined by a fixed multidisciplinary team (MDT) based on anatomical, functional, and oncological characteristics. The specific criteria are as follows: 1. Insufficient future liver remnant (FLR): For cirrhotic patients, the FLR accounts for < 40% of the standard liver volume; for non-cirrhotic patients, this proportion is < 30%. 2. R0 resection unattainability: Despite adequate FLR, unresectability is determined if the tumor’s size, number, distribution, vascular invasion, and other factors prevent complete resection with a safe margin. All patients were treated with a minimum of two cycles of combination therapy involving PD-1/PD-L1 inhibitors and Tyrosine kinase inhibitors (TKIs) (chemotherapy could be included). TKIs included lenvatinib and donafenib; PD-1/PD-L1 inhibitors included camrelizumab, tislelizumab, toripalimab, pembrolizumab, envafolimab, and durvalumab. The detailed conversion therapy regimens for unresectable CCA are comprehensively presented in **Supplementary Table 1**. Chemotherapy regimens were gemcitabine plus cisplatin or oxaliplatin. Inclusion criteria: (1) Aged > 18 years and < 80 years. (2) Received PD-1/PD-L1 inhibitor plus TKIs-based combination conversion therapy. (3) Underwent surgical resection or continued the original systemic treatment regimen after successful conversion. Exclusion criteria: (1) Residual or recurrent CCA. (2) Patients with concurrent other tumors. (3) Missing important clinical data or follow-up data. Evaluation of tumor response was performed based on RECIST 1.1 (19). This study was approved by the Ethics Committee of Hunan Provincial People’s Hospital (Approval No.: [2023]-156).

### Data collection

In accordance with the inclusion and exclusion criteria, data of enrolled patients were collected, including gender, age, carbohydrate antigen 19-9 (CA19-9) levels before conversion therapy, tumor size and number, tumor response, duration of conversion therapy, time of surgery, blood loss, and surgical complications. Surgical complications were graded based on the Clavien et al. (20) classification system.

### Assessments and endpoint indicators

For tumor evaluation, computed tomography (CT) or magnetic resonance imaging (MRI) was performed at 2–4 cycle intervals, with positron emission tomography (PET) selectively adopted when clinically indicated. The surgical indications after successful conversion were as follows: 1. Definite tumor shrinkage or achievement of partial response (PR) confirmed by CT or MRI, lasting for at least 1 month; 2. Feasibility of R0 resection evaluated by the MDT; 3. Stable tumor marker levels; 4. Adequate future liver remnant (FLR); 5. No contraindications to surgical resection. Before conversion surgery, PD-1/PD-L1 inhibitors and TKIs were discontinued for at least 2 weeks (chemotherapeutic agents for at least 3 weeks). For patients in the non-surgery group, all of them were confirmed to meet the resectability criteria through MDT discussions. After being fully informed of the two treatment options—surgical resection and continued systemic therapy—the patients and their families opted for continued systemic therapy after comprehensive consideration, mainly due to concerns regarding treatment costs and therapeutic efficacy. The study endpoints included PFS (referring to the interval from when conversion therapy begins to tumor progression, last follow-up, or death); RFS (defined as the duration from surgical resection to tumor recurrence or last follow-up), and OS (referring to the period from the initiation of conversion therapy to death or last follow-up).

### Statistical analysis methods

For statistical comparisons between groups, we employed Student’s t-test, chi-square test (χ² test), and Fisher’s exact test. The Kaplan-Meier method was used to plot survival curves, with the COX regression model utilized to assess factors impacting patient outcomes.

## Results

### Patient baseline characteristics

**Figure 1:**
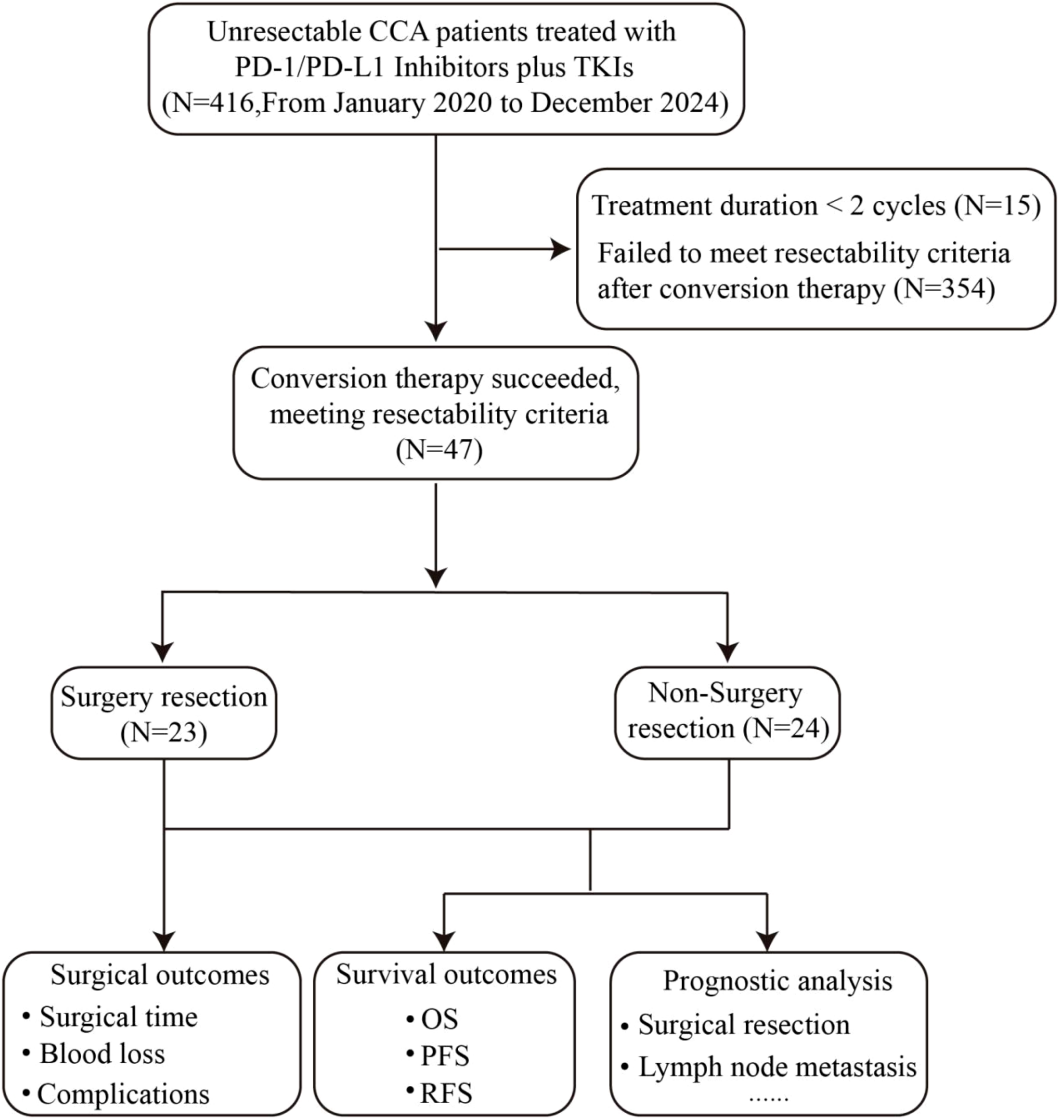
Enrollment case screening flow chart. CCA, cholangiocarcinoma; OS, Overall survival; PFS, progression-free survival; RFS, recurrence-free survival.

Between June 2020 and December 2024, we identified 416 patients with initially unresectable CCA in total. Among them, 15 patients received fewer than 2 treatment cycles and were excluded from the subsequent analysis; of the remaining 401 eligible patients, 47 achieved resectability after conversion therapy, corresponding to a conversion rate of 11.7% (47/401) in the eligible cohort. Ultimately, 23 patients underwent surgical treatment, and 24 patients opted to continue systemic treatment (**Figure 1**). Before initiation of conversion therapy, baseline data and disease characteristics were comparable between the two groups (**Table 1**). For the entire study cohort, the median age was 59 years (44–76 years), and 28 males accounted for 59.6%. 38 patients (80.9%) had an Eastern Cooperative Oncology Group Performance Status (ECOG PS) score of 0, and nine patients had a score of 1. Among them, 24 patients (51.1%) had elevated carbohydrate antigen 19-9 (CA19-9) levels, 14 patients (28.8%) had multiple tumor lesions, and 24 patients (51.1%) had lymph node metastasis. There were no significant differences between the two groups in terms of tumor subtypes, chemotherapy combination status, treatment line (first-line vs. ≥second-line), and conversion therapy course.

**Table 1:** Baseline characteristics of the surgical group and non-surgical group.

Characteristics	NR	SR	P value
n	24	23	
Sex, n (%)			0.312
Female	8 (33.3%)	11 (47.8%)	
Male	16 (66.7%)	12 (52.2%)	
Age(years), mean ± sd	61.3 ± 8.7	57.5 ± 6.7	0.104
ECOG PS, n (%)			0.502
0	18 (75.0%)	20 (87.0%)	
1	6 (25.0%)	3 (13.0%)	
CA19-9(U/ml), n (%)			0.664
≤37	11 (45.8%)	12 (52.2%)	
>37	13 (54.2%)	11 (47.8%)	
Tumor number, n (%)			0.170
single	19 (79.2%)	14 60.9%)	
multiple	5 (20.8%)	9 (39.1%)	
Lymph node metastasis, n (%)			0.109
Yes	15 (62.5%)	9 (39.1%)	
No	9 (37.5%)	14 (60.9%)	
Subtypes, n (%)			1.000
iCCA	22 (91.7%)	22 (95.7%)	
pCCA	2 (8.3%)	1 (4.3%)	
Treatment course, median (IQR)	4.5 (3, 6)	6 (3.5, 7)	0.273
Treatment line, n (%)			0.312
1	15 (62.5%)	11 (48.7%)	
2	9 (37.5%)	12 (52.2%)	
Chemotherapy Combination, n (%)			0.307
Yes	17 (70.8%)	13 (56.5%)	
No	7 (29.2%)	10 (43.5%)	

### Tumor outcomes and subsequent treatment regimens

**Figure 2:**
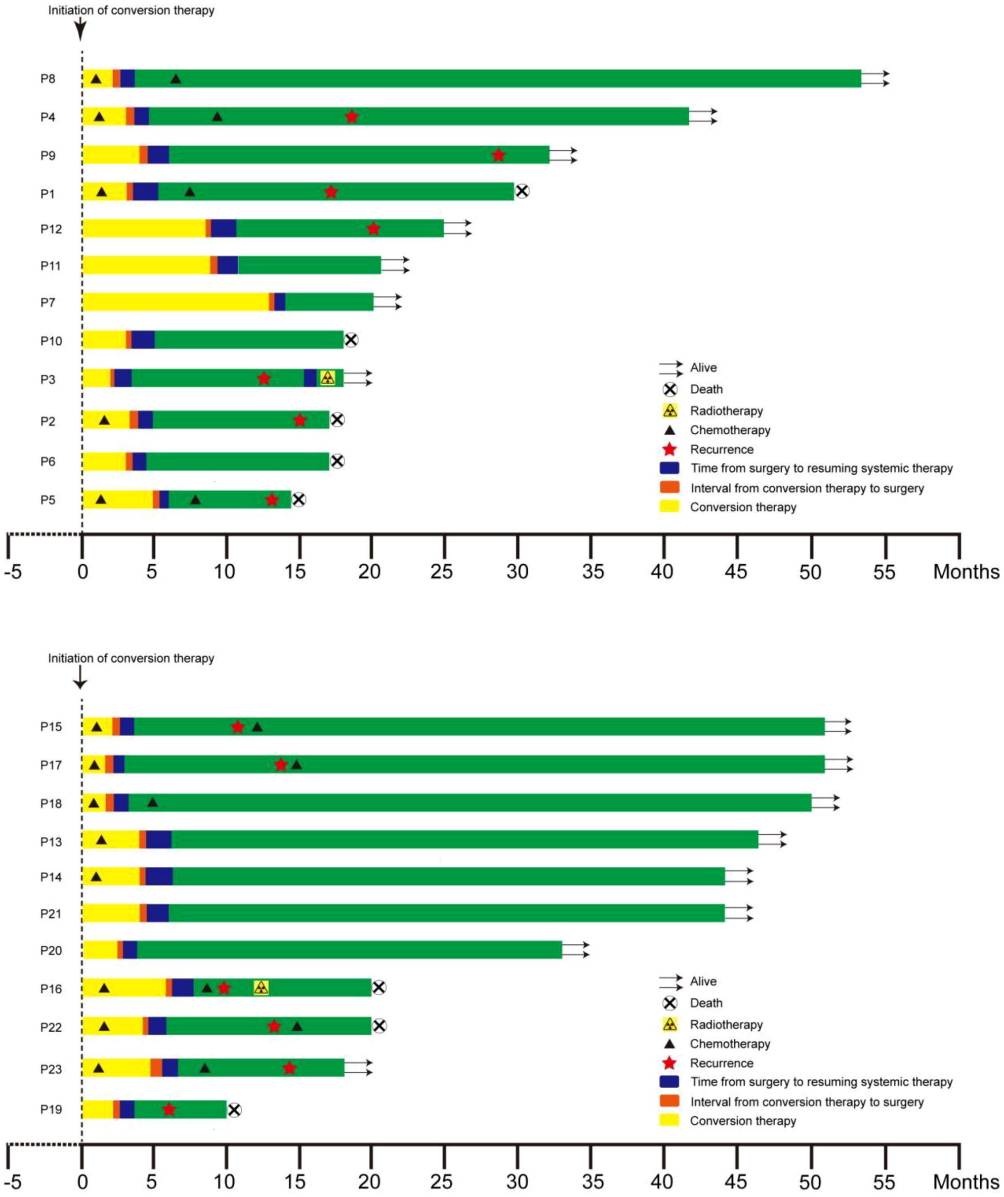
Swimmer plot of treatment courses in patients of the surgical group.

A total of 47 patients achieved at least PR and were evaluated as resectable by the MDT. Patients in the SR were deemed eligible for surgery after MDT discussion and underwent surgical treatment following a drug withdrawal period of at least 2 weeks for TKIs and PD-1/PD-L1 inhibitors (and at least 3 weeks for chemotherapeutic agents). Patients in the NR continued the previous systemic treatment regimen. By the cutoff date of follow-up, in the NR, 2 patients had continued tumor shrinkage, 2 patients maintained stability after achieving PR, 9 patients experienced disease progression, 10 patients developed intrahepatic metastasis, and 1 patient developed bone metastasis. Patients in the SR underwent surgery after MDT re-evaluation upon meeting the surgical criteria. The average operation time was 239 ± 58 minutes, with an average blood loss of 204 ± 104 milliliters. Among them, 14 patients underwent laparoscopic hepatectomy and nine underwent open surgery. A total of 21 patients (91.3%) achieved R0 resection. Pathological results showed microvascular invasion in eight patients. 7 patients developed grade II complications, all of which were cured with conservative treatment; no complications above grade IIIa occurred, and there were no surgery-related deaths (**Table 2**). By the cutoff date of follow-up, 12 patients had intrahepatic recurrence, 1 patient had lung metastasis, and another 10 patients showed no recurrence to date. After surgery, 1 patient received additional radiotherapy, while another underwent reoperation combined with radiotherapy. 10 patients were administered chemotherapy alongside PD-1/PD-L1 inhibitors plus TKIs (**Figure 2**).

**Table 2:** Treatment prognostic outcomes and perioperative data of the surgical group.

Parameter	SR (23)	NR (24)	P
Median OS (months)	Not Reached	28.5	
Median PFS (months)	19	13.5	
OS rate (%)			0.026
1-year	95.7%	91.7%	
3-year	68.5%	18.9%	
PFS rate (%)			
1-year	87.0%	61.6%	0.025
3-year	26.1%	8.9%	
RFS rate (%)			
1-year	54.7%	–	
3-year	29.3%	–	
Surgical approaches			
Laparoscopy	14	–	
Open Surgery	9	–	
Operation time (min)	239 ± 58	–	
Blood Loss (ml)	204 ± 104	–	
R0 resection rate (n,%)	21 (91.3%)	–	
Microvascular Invasion (n,%)	8 (34.8%)	–	
Postoperative complications (Clavien–Dindo II)	7 (30.4%)	–	

### Prognostic comparison between the SR and the NR

**Figure 3:**
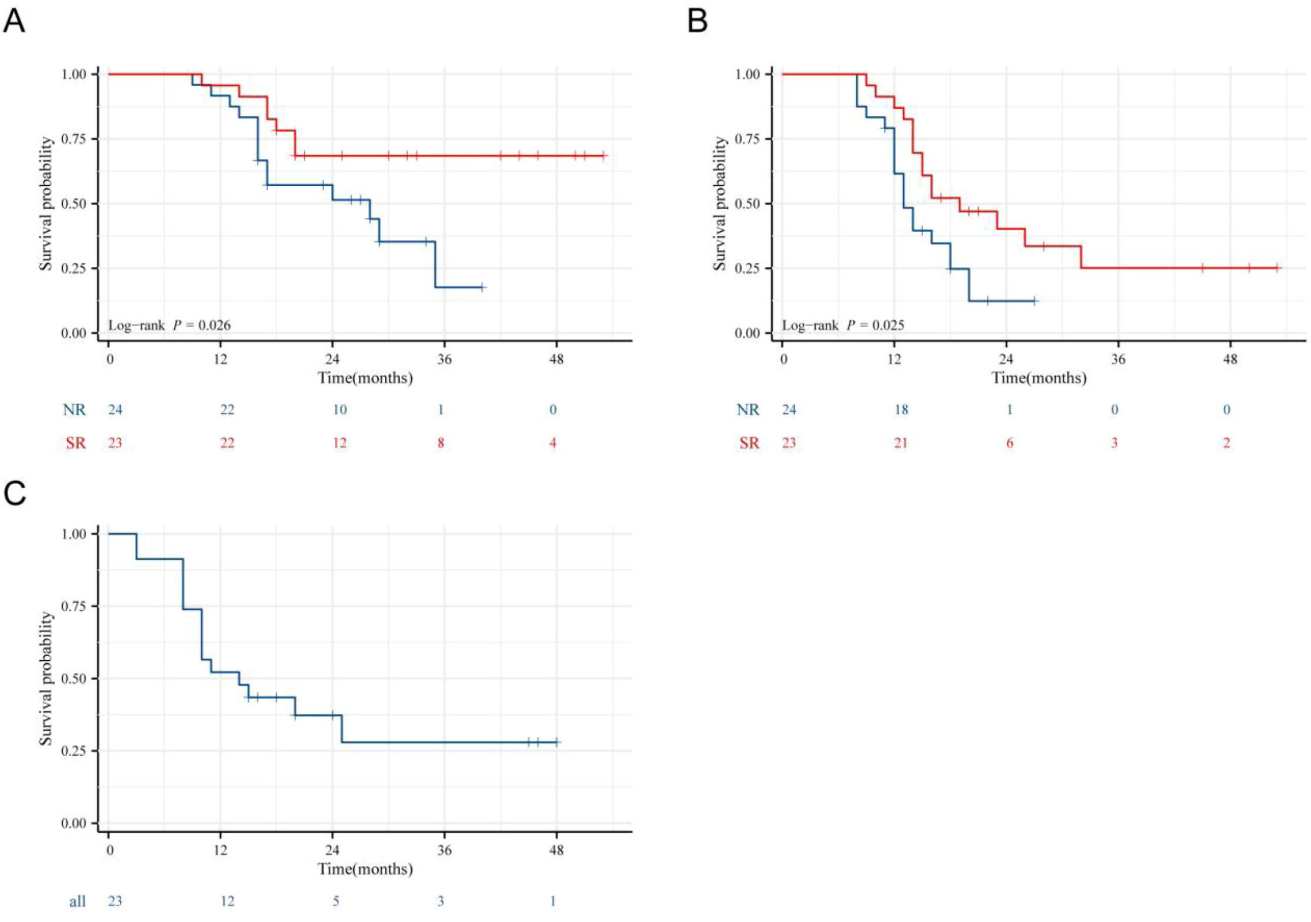
**(A–C)** OS, PFS, and RFS curves of the surgical group and non-surgical group. **(A)** OS curves of the surgical group vs. non-surgical group (P = 0.026); **(B)** PFS Curves of the surgical group vs. non-surgical group (P = 0.025); **(C)** RFS Curve of the surgical group.

The follow-up period ended in September 2025, with all patients having a follow-up time of 20 months (9–52 months). The SR had a median OS that was not reached. Its 1-, 2-, and 3-year OS rates were 95.7%, 68.5%, and 68.5%, respectively. In contrast, the NR had a median OS of 28.5 months, with 1-, 2-, and 3-year OS rates of 91.7%, 51.9%, and 18.9%. The SR had a significantly longer OS than the NR (P = 0.026) (**Figure 3A**). SR had a median PFS of 19 months. Its 1-, 2-, and 3-year PFS rates were 87.0%, 40.2%, and 25.2%, respectively—all significantly higher than those in the NR. For the NR, the median PFS was 13.5 months, with corresponding 1-, 2-, and 3-year PFS rates of 61.6%, 12.4%, and 12.4% (P = 0.025) (**Figure 3B**). The 1-, 2-, and 3-year recurrence-free survival (RFS) rates in the SR were 54.7%, 39.0%, and 29.3%, respectively (**Figure 3C**).

### Risk factors for prognosis in conversion therapy patients

A COX regression model was used to analyze the factors influencing the prognosis of patients receiving conversion therapy. The variables included surgical resection status, gender, age, ECOG PS score, CA19–9 level, number of tumor lesions, lymph node metastasis status, and number of conversion therapy cycles. The final results showed that surgical resection was a protective factor for OS and PFS of patients, while lymph node metastasis was a risk factor affecting patients’ PFS (**Tables 3**, **4**).

**Table 3:** Univariate and multivariate analyses of factors influencing OS in patients undergoing conversion therapy.

Characteristics	Total (N)	Univariate analysis		Multivariate analysis	
		Hazard ratio (95% CI)	P value	Hazard ratio (95% CI)	P value
Group	47				
NR	24	Reference		Reference	
SR	23	0.366 (0.146 - 0.918)	0.032	0.366 (0.146 - 0.918)	0.032
Sex	47				
Male	28	Reference			
Female	19	1.363 (0.578 - 3.213)	0.479		
Age(years)	47	1.029 (0.975 - 1.085)	0.301		
ECOG PS	47				
0	38	Reference			
1	9	2.094 (0.803 - 5.463)	0.131		
CA19-9(U/ml)	47				
>37	24	Reference			
≤37	23	0.844 (0.358 - 1.991)	0.699		
Tumor number	47				
single	33	Reference			
multiple	14	0.498 (0.167 - 1.486)	0.211		
Lymph node metastasis	47				
No	23	Reference			
Yes	24	2.076 (0.856 - 5.036)	0.106		
Treatment course	47	0.904 (0.749 - 1.092)	0.295		

**Table 4:** Univariate and multivariate analyses of factors influencing PFS in patients undergoing conversion therapy.

Characteristics	Total (N)	Univariate analysis		Multivariate analysis	
		Hazard ratio (95% CI)	P value	Hazard ratio (95% CI)	P value
Group	47				
NR	24	Reference		Reference	
SR	23	0.451 (0.223 - 0.913)	0.027	0.469 (0.229 - 0.959)	0.038
Sex	47				
Male	28	Reference			
Female	19	0.714 (0.350 - 1.456)	0.354		
Age(years)	47	0.994 (0.950 - 1.040)	0.799		
ECOG PS	47				
0	38	Reference			
1	9	0.980 (0.375 - 2.563)	0.967		
CA19-9(U/ml)	47				
>37	24	Reference			
≤37	23	1.199 (0.600 - 2.396)	0.606		
Tumor number	47				
single	33	Reference			
multiple	14	1.447 (0.709 - 2.952)	0.310		
Lymph node metastasis	47				
No	23	Reference		Reference	
Yes	24	2.267 (1.117 - 4.598)	0.023	2.186 (1.065 - 4.489)	0.033
Treatment course	47	0.929 (0.811 - 1.064)	0.289		

## Discussion

CCA, a common malignant tumor of the biliary tract, is characterized by an insidious onset and high invasiveness. Approximately 65% of patients present with locally advanced or metastatic disease at initial diagnosis, thus losing the opportunity for curative surgery (2). Gemcitabine plus cisplatin is the standard conventional chemotherapy regimen, but the median OS is only about 12 months, which is far from meeting clinical needs (21). In recent years, the emergence of ICIs and TKIs therapy has brought new hope to patients with advanced CCA. Their synergistic anti-tumor mechanisms have significantly improved the tumor downstaging rate of unresectable CCA, opening up a new avenue for conversion therapy (22, 23). However, for patients who achieve resectable criteria after successful conversion, targeted evidence focusing on the optimal subsequent strategy between “surgical resection vs. continuous systemic therapy alone” remains lacking. Furthermore, most existing studies are confounded by chemotherapeutic agents, which makes it hard to clarify surgery’s true therapeutic value. This evidence gap significantly undermines the scientific basis of clinical decision-making.

We conducted a retrospective analysis of 47 CCA patients who achieved successful conversion therapy between January 2020 and December 2024. These patients were treated with PD-1/PD-L1 inhibitors combined with TKIs or other therapeutic regimens. Our data revealed that surgical resection was an independent protective factor for both OS and PFS in this cohort. Importantly, our findings validate the survival advantage of conversion surgery in CCA treatment and provide key evidence-based medicine for clinical decision-making in this patient population. A study (24) found that advanced CCA patients who successfully converted to resectable disease with selective internal radiation therapy plus chemotherapy had improved prognosis after subsequent surgery. Specifically, their 1-year OS rate reached 75%, and the 2-year OS rate was 45%. In comparison, results from a recent CCA study (12) based on immunotherapy conversion showed that the 1- and 2-year OS rates in the surgical group were 92.3% and 83.9%, respectively, which were significantly higher than those in the non-surgical group. The above studies are consistent with our findings. The notable survival benefit of conversion surgery stems from overcoming the inherent limitations of systemic therapy alone. While PD-1/PD-L1 inhibitor-based conversion regimens combined with TKIs can curb tumor growth and achieve tumor downstaging in some patients, they still cannot fully eradicate residual tumor cells. These cells are often the “root cause” of subsequent drug resistance and disease progression. Tumor cells can escape immune surveillance via immune editing mechanisms. Long-term use of immunotherapy is prone to inducing T-cell exhaustion; exhausted T cells highly express immunosuppressive markers such as PD-1 and TIGIT, lose anti-tumor activity, and thereby lead to therapeutic resistance (25). This resistance mechanism cuts into the long-term effectiveness of immunotherapy and boosts the risk of subsequent disease progression in patients. Additionally, targeted therapy is inherently “target-dependent”—meaning it can’t cover all tumor cell subsets. Residual non-target-dependent cells may go on proliferating, which in turn leads to recurrence and drug resistance (26). Conversion surgery, by thoroughly removing residual lesions, forms a synergistic effect of “systemic control + local radical cure” with preoperative conversion treatment, fundamentally eliminating this risk and ultimately maximizing survival benefits. This may also be the key reason why the 3-year OS rate in the SR was nearly four times higher than that in the NR. Based on existing research evidence, our findings are highly consistent with previous studies on conversion surgery for CCA, while highlighting the unique advantages of the “PD-1/PD-L1 inhibitors plus TKIs” conversion modality. During the chemotherapy era, a multicenter retrospective study by Noji et al (27) showed that patients with initially unresectable biliary tract cancers who underwent surgical treatment after chemotherapy-induced conversion achieved significantly better OS than those treated with chemotherapy alone. However, chemotherapy-based conversion regimens have low conversion efficiency—less than 20%—which has held back their clinical use. In contrast, our study used a combined conversion regimen of PD-1/PD-L1 inhibitors plus TKIs. The SR’s 3-year OS rate hit 68.5%, a result that notably improves survival outcomes for patients with advanced CCA.

Our multivariate analysis revealed that surgical resection serves as an independent protective factor for both OS and PFS in patients with advanced CCA. Even if patients had baseline metastases (such as those with possible lymph node metastasis in this study), surgical resection could still bring significant survival benefits as long as resectable criteria were achieved after conversion therapy. Our study suggests that in the era of immuno-targeted therapy, treatment decisions for CCA shouldn’t be confined solely to traditional tumor staging. Instead, they should be based on individualized assessments of patients’ tumor status following conversion therapy. For patients who meet resectable criteria, surgical treatment should be actively recommended. Meanwhile, our study identified lymph node metastasis as an independent risk factor for PFS, and this result provides important evidence for clinical risk stratification and individualized management. Lymph node metastasis usually indicates that the tumor has certain invasiveness and metastatic potential; even if macroscopic tumor downstaging is achieved through conversion therapy, micrometastases or residual tumor cells may still exist (28). These patients have a higher risk of postoperative recurrence and require more intensive postoperative management strategies. Based on existing research evidence (29), for patients undergoing conversion surgery with concurrent lymph node metastasis, prolonged duration of postoperative immunotargeted therapy or combination with local therapy (such as radiotherapy) may be considered to further control the risk of local recurrence. Moreover, more frequent follow-up surveillance (e.g., enhanced CT/MRI scans and tumor marker testing every 2–3 months) facilitates early recurrence detection and timely clinical intervention, ultimately optimizing PFS. However, PFS was defined as the time from conversion therapy initiation to tumor progression, last follow-up, or death, with inherent limitations: it fails to isolate surgery’s independent effect on tumor control and cannot distinguish progression due to systemic therapy resistance or incomplete resection, resulting in poor specificity for evaluating surgical efficacy. In contrast, RFS starts at surgical resection and focuses on tumor recurrence as the core endpoint. It directly reflects surgical curative effect and long-term tumor control, thus serving as a key indicator for assessing conversion surgery value.

In this study, the 1-, 2-, and 3-year RFS rates in the surgical group were 54.7%, 39.0%, and 29.3%, respectively, with an R0 resection rate of 91.7%. Two patients underwent R1 resection and developed recurrence and intrahepatic metastasis three months postoperatively. Based on these findings, we recommend that if the surgical margin cannot be determined intraoperatively, intraoperative frozen section examination should be performed to confirm whether the margin is negative. For patients in whom R0 resection is not feasible due to insufficient residual liver volume or invasion of major blood vessels identified intraoperatively, titanium clips can be used for marking, followed by postoperative radiotherapy plus comprehensive therapy. Regular follow-up should be conducted to monitor tumor marker levels, so as to detect tumor changes in a timely manner, adjust treatment regimens, and prolong patient survival. No complications above grade IIIa occurred in 23 patients in the surgical group of this study, and there were no surgery-related deaths. This indicates that conversion surgery has favorable safety and feasibility. This conclusion is consistent with the safety data from existing relevant studies: Wang et al (18) reported that the incidence of Clavien-Dindo grade ≥3 complications after conversion surgery following PD-1/PD-L1 inhibitors combination therapy was only 7.7%, with no surgery-related deaths; in a study on conversion therapy with “Lenvatinib plus PD-1 inhibitor” conducted by Zhang et al (30), the postoperative complication rate in surgical patients was also controlled at a low level.

While our study yields meaningful findings, it still has several limitations. First, this was a single-center retrospective study, so selection bias is unavoidable. For example, decisions to proceed with surgery might be shaped by factors such as patients’ physical condition and treatment preferences. Additionally, the single-center sample lacks strong representativeness, and the generalizability of our results will require further validation through multi-center prospective studies.

## Data Availability

The original contributions presented in the study are included in the article/**Supplementary Material**. Further inquiries can be directed to the corresponding author.
