# Supplementary material for: Surgery vs. non-surgery for advanced cholangiocarcinoma post-conversion therapy with PD-1/PD-L1 inhibitors plus TKIs
Source: Front Immunol. 2026 Jan 29;17:1753437. doi: 10.3389/fimmu.2026.1753437 (PMC12894232; doi:10.3389/fimmu.2026.1753437)
Supplement: Supplementary file 1 [file Table1.docx]

****Supplementary Table 1**** Detailed Conversion Therapy Regimens for Unresectable CCA

| Treatment Category | Specific Agents | Administration Frequency |
| --- | --- | --- |
| **PD-1 inhibitors** | Camrelizumab | every 3 weeks |
|  | Tislelizumab | every 3 weeks |
|  | Toripalimab | every 3 weeks |
|  | Pembrolizumab | every 3 weeks |
| **PD-L1 inhibitors** | Envafolimab | weekly |
|  | Durvalumab | every 3 weeks |
| **TKIs** | Lenvatinib | once daily, oral administration |
|  | Donafenib | once daily, oral administration |
| **Chemotherapy regimens** | GC (Gemcitabine + Cisplatin) | 21-day cycle: Gemcitabine 1000 mg/m² (intravenous infusion on Days 1 and 8), Cisplatin 25 mg/m² (intravenous infusion on Days 1 and 8) |
|  | GEMOX (Gemcitabine + Oxaliplatin) | 21-day cycle: Gemcitabine 1000 mg/m² (intravenous infusion on Days 1 and 8), Oxaliplatin 100 mg/m² (intravenous infusion on Day 1) |

First-line: Camrelizumab+Lenvatinib+GEMOX; Pembrolizumab+Lenvatinib+GC; Toripalimab+Lenvatinib+GEMOX; Durvalumab+Lenvatinib+GC.

≥second-line: Tislelizumab+Lenvatinib; Pembrolizumab+Lenvatinib; Envafolimab+Lenvatinib.
